# Vivax Malaria and the Potential Role of the Subtelomeric Multigene *vir* Superfamily

**DOI:** 10.3390/microorganisms10061083

**Published:** 2022-05-24

**Authors:** Youn-Kyoung Goo

**Affiliations:** Department of Parasitology and Tropical Medicine, School of Medicine, Kyungpook National University, Daegu 41944, Korea; kuku1819@knu.ac.kr; Tel.: +82-53-420-4882

**Keywords:** *Plasmodium vivax*, *vir* gene, severe vivax malaria, cytoadherence, immune response

## Abstract

Vivax malaria, caused by *Plasmodium vivax*, remains a public health concern in Central and Southeast Asia and South America, with more than two billion people at risk of infection. Compared to *Plasmodium falciparum*, *P. vivax* is considered a benign infection. However, in recent decades, incidences of severe vivax malaria have been confirmed. The *P. falciparum* erythrocyte membrane protein 1 family encoded by *var* genes is known as a mediator of severe falciparum malaria by cytoadherence property. Correspondingly, the *vir* multigene superfamily has been identified as the largest multigene family in *P. vivax* and is implicated in cytoadherence to endothelial cells and immune response activation. In this review, the functions of *vir* genes are reviewed in the context of their potential roles in severe vivax malaria.

## 1. The Biological and Pathophysiological Aspects of Vivax Malaria

Malaria is one of the most serious vector-borne infections and is caused by the *Plasmodium* sporozoites from infected *Anopheles* mosquitoes to humans [1]. According to the World Health Organization, half of the global population is at a risk of malaria. In 2020, 241 million cases of malaria were reported, with a death toll of 627,000 [2]. Among more than 200 *Plasmodium* species known to infect vertebrates, only five cause malaria in humans: *P. falciparum*, *P. vivax*, *P. ovale*, *P. malariae*, and *P. knowlesi*. Together, *P. falciparum* and *P. vivax* are responsible for approximately 90% of malaria cases worldwide. While *P. falciparum* infection is associated with high mortality, reports of severe vivax malaria have increased over the last decade [3,4]. 

A fundamental difference between *P. vivax* and *P. falciparum* is the ability of dormant *P. vivax* hypnozoites to reactivate. Furthermore, *P. vivax* preferentially infects reticulocytes, which seems to limit its reproductive capacity, whereas *P. falciparum* invades red blood cells (RBCs) of all ages, which allows it to rapidly reach a high parasite burden without treatment. Following *P. vivax* infection, parasitemia rarely reaches over 2% of circulating RBCs, and severe vivax malaria manifestations are not always related to high parasite burdens. This is in contrast to severe malaria caused by *P. falciparum* infection, in which hyperparasitemia (more than 5% parasitemia) is one of manifestations of severe cases [5,6]. In addition, a protective immune response of immune cells such as natural killer cells and specific antibody production to *P. falciparum* parasites was related to an induction of severe falciparum malaria [7,8,9,10]. Compared to *P. falciparum*, the pathogenicity inducing severe vivax malaria is less well-defined, thus the mechanism is needed to be elucidated. 

Severe vivax malaria is associated with severe anemia, respiratory distress, acute lung injury, splenic injury, acute renal failure, coma, low birth weight, and malnutrition [11,12]. Severe anemia is induced by the destruction and invasion of reticulocytes by *P. vivax* parasites and the increased fragility of infected and non-infected RBCs. Cytokine-related dyserythropoiesis may also contribute to anemia [13]. High cytokine production by *P. vivax* parasites induces organ-specific inflammation and endothelial dysfunction in the lungs, spleen, kidneys, and utero-placenta, which ultimately results in severe vivax malaria [14,15,16]. Although lipid- or parasite-derived molecules have been described as causes of inflammation and fever in vivax malaria, the underlying mechanisms have not been elucidated [17,18,19].

While *P. vivax* is infrequently associated with severe malaria in pregnant women compared to *P. falciparum*, it is known to be responsible for maternal anemia and reduced birth weight [20]. Malnutrition is also associated with vivax malaria, especially during early childhood, and is likely to be induced by deleterious catabolic responses related to chronic relapsing-remitting inflammation [21,22,23]. Other factors associated with severe malaria include population characteristics (host genetics, geographic and social factors, age, and *P. vivax* chloroquine resistance) and virulence factors [11]. The lack of Duffy antigen receptors in the West African population and prevalence of α^+^-thalassemia contribute to the morbidity of vivax malaria [24,25,26,27]. In addition, a high prevalence of chloroquine-resistant *P. vivax* is considered an important contributor to the incremental risk of severe vivax anemia [28]. Virulence factors at the molecular level are yet to be described; however, virulence molecules have been a focus of genomic and transcriptomic studies of *P. vivax*-infected hosts with different phenotypes and disease severities [29]. In *P. falciparum*, the var gene family encoding *P. falciparum* erythrocyte membrane protein 1 (PfEMP1) family performs a key role in antigenic variation and cytoadherence [30]. The similar multigene superfamily in *P. vivax*, *vir*, has been studied as an important multigene superfamily in the pathogenesis of vivax malaria and resulting host immune activation [31,32].

## 2. The Subtelomeric *vir* Multigene Superfamily

The *Plasmodium* species contain multigene families in the telomeric and subtelomeric regions of their chromosomes, which code for variant surface antigens (VSAs). The *Plasmodium* interspersed repeat (*pir*) genes comprise the largest multigene family identified to date, which includes *var* in *P. falciparum*; *vir* in *P. vivax*; *kir* in *P. knowlesi*; and *cir*, *bir*, and *yir* in the three rodent malaria parasites *P. chabaudi*, *P. berghei*, and *P. yoelli,* respectively [33,34,35,36,37,38,39]. Gene structural analysis and protein structural prediction on a whole-genome scale demonstrated that *vir* genes are closely related to *kir* genes but not to *var* genes [40]. Moreover, annotation analysis of the genome sequences of *Plasmodium* species revealed that *vir* genes were much more abundant than *var* genes [36] (Table 1). 

**Figure 1:**
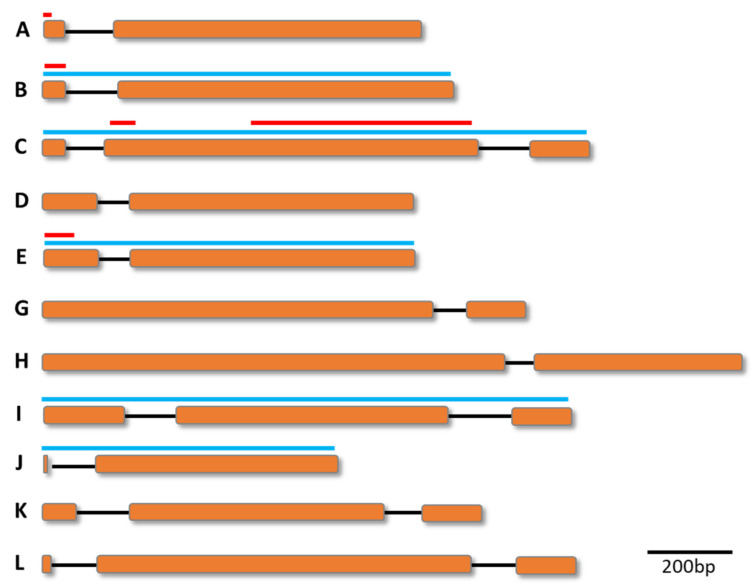
Schematic representation of gene models from the different subfamilies, A–L, of the *Plasmodium vivax* subtelomeric vir multigene superfamily. Exons are presented as orange boxes and introns as lines. Red bars above subfamilies A, B, C, and E showed the relative position of recombinant proteins or peptides used for an immune response assay. Blue bars above subfamilies B, C, E, I, and J showed the position of gene diversity analysis. Scale in nucleotides (base pairs, bp).

The *vir* genes range from 156–3434 bp in size and each gene has one to five exons. The first exon lacks the signal peptide sequences, and the second exon is highly polymorphic and contains the predicted transmembrane domain and conserved cysteine residues. The third exon is uniform in size, encoding for the putative cytosolic domain. The region between the second and the third exons is well-conserved [41,42]; 600–1000 *vir* genes per haploid genome in six subfamilies (A–F) were initially identified in *P. vivax* isolates obtained from patients with vivax malaria [34], whereas 346 *vir* genes in 12 subfamilies (A–L) were annotated in the *P. vivax* Sal-I genome [35] (Figure 1). More recently, over 1200 *vir* genes in 27 subfamilies were identified in samples obtained from infected patients, in which a more accurately annotated assembly with fragmentation reduced to <250 scaffolds, and the inclusion of extended subtelomeric regions of chromosomes resulted in improved results over the Sal I reference genome [36] (Table 1). Although these previous studies showed a similar distribution of *vir* genes among subfamilies and isolates, inconsistent numbers of subfamilies and *vir* genes were observed; therefore, it is necessary to establish robust reference sequences and accurate discrimination criteria to generate a standard classification of *vir* genes.

Confocal laser scanning microscopy with immune sera against a conserved peptide sequence from subfamily D demonstrated that VIR proteins localize to the surface of infected reticulocytes. In silico analysis of protein domains and secondary structures from parasite sequences obtained directly from patients revealed that Vir subfamily A contains 2TM domains, similar to the Pfmc-2TM multigene family. Additionally, subfamily D was found to be related to the SURFIN subtelomeric *P. falciparum* multigene family [34]. Notably, SURFIN proteins are located at the surface of merozoites and infected RBCs, whereas Pfmc-2TM proteins are located mainly at Maurer’s clefts [43,44]. Furthermore, previous data showed that only 160 VIR proteins possess a *Plasmodium* export element (PEXEL)-like motif, which functions in the intracellular transport of malarial proteins to the surface of infected red blood cells and cytosol [34]. Together, these data suggest that VIR proteins may have various localizations depending on subfamilies, on subcellular compartments, or the surface membrane. Therefore, further studies are required to determine the precise functions of VIR proteins in each subfamily. 

## 3. Immune Response Induced by VIR Proteins

In *P. falciparum*, each parasite has approximately 60 *var* genes in its genome but expresses only one *var* gene at a time; this phenomenon is known as mutually exclusive expression. Unlike the *var* genes of *P. falciparum*, *vir* genes do not undergo allelic exclusive expression, and a vast repertoire of *vir* genes is abundantly expressed in isolates at any given time. In addition, antibody detection levels in immune sera between first-infected and multiple-infected patients were not significantly different [45]. This long-lasting antibody response against VIR proteins without clonal expression would be of great advantage in developing a vivax malaria vaccine using VIR proteins to overcome its high genetic diversity [46,47,48]. 

The acquisition of natural immunity to VIR proteins was evaluated using recombinant proteins generated in wheat germ cell-free and *Escherichia coli* expression systems or synthetic peptides (Table 2) [19,32,45,49]. Glutathione-S-transferase (GST)-tagged VIR proteins of subfamilies A–E were used to reveal similar immune reactions between first- and multiple-infected patients with vivax malaria [45]. In addition, soluble GST-tagged VIR proteins and synthetic peptides have been used in immune-epidemiological studies in vivax malaria endemic areas in Brazil, South Korea, Columbia, Guatemala, India, and Papua New Guinea [19,32,49]. Approximately 50% of the population presented antibodies against VIR proteins (IgM or IgG) during vivax malaria; this was similar to the frequency of apical membrane antigen-1 (AMA-1) antibodies but lower than that of the 19kDa fragment of merozoite surface protein 1 (MSP1-19). The antibody response of VIR-C2 and PvLP2 (a synthetic peptide) presented in the higher percentile (over 15%) of the tested population compared to that of other recombinant VIR proteins (approximately 10%) [19,49], and VIR25 was the most broadly recognized antigen in malaria-endemic countries [32]. Moreover, a low proliferative response by peripheral blood mononuclear cells to variant VIR proteins and synthetic peptides was observed, which was similar to the results for MSP1-19 in *P. falciparum* and *P. vivax* [19,49]. The natural acquisition of cellular immunity was also observed in studies of VIR-C2 and PvLP2, in which the cytokines IL-2, IL-6, IL-10, and TNF acted to produce effector helper cells for cell expansion in acute malaria [19,49]. This promoted T-cell differentiation to trigger cellular memory, and regulatory immune responses showed higher levels of secreted cytokines [50,51]. Interestingly, antibody levels of VIR2 and VIR24 were positively associated with birth weight in a study on vivax malaria in pregnant women, which was the first report evaluating the association between the host immune response to VIR proteins and malaria outcomes [32]. To date, there have been limited studies reporting on naturally acquired immune response-induced VIR proteins in field populations from vivax malaria-endemic areas. Extensive prospective longitudinal or cohort epidemiological studies of naturally acquired immunity against different antigens in *P. falciparum*, as well as against MSP1 and the Duffy-binding protein of *P. vivax* previously reported, may help determine the roles of these proteins in malaria protection and/or reduced risk of infection [52,53,54]. Therefore, larger prospective cohort studies are needed to specifically address whether VIR proteins are targets of protective immunity and have the potential to be vaccine candidates.

**Table 2:** VIR proteins and synthetic peptides studied previously.

Subfamily *	Name	Evaluated Aspects	References
A	VIR-A4	IgG and IgM, IgG subclass, PBMC proliferation	[49]
B	VIR-B10	IgG and IgM, IgG subclass, PBMC proliferation	[49]
	VIR21	Genetic diversity in India and South Korea	[46,47,48]
C	VIR-C1	IgG and IgM, IgG subclass, PBMC proliferation	[19,49]
	VIR-C2	IgG and IgM, IgG subclass, PBMC proliferation, levels of cytokine and growth factor	[19,49]
	VIR-C16	IgG and IgM	[49]
	VIR4	Genetic diversity in India and South Korea	[46,47,48]
	VIR14	IgG and IgM, IgG subclass, spleen-dependent cytoadherence	[19,32]
	VIR25	IgG and IgM, IgG subclass, PBMC proliferation, levels of cytokine and growth factor	[19,32]
E	VIR-E5	IgG and IgM	[49]
	VIR-E8	IgG and IgM	[49]
	VIR-E	IgG and IgM, cytoadherence to Chinese hamster ovary cells	[31]
	VIR2	IgG and IgM, IgG subclass	[19,32]
	VIR5	IgG and IgM	[32]
	VIR12	Genetic diversity in India and South Korea	[46,47,48]
	VIR24	IgG and IgM	[32]
I	VIR27	Genetic diversity in India and South Korea	[46,47,48]
J	VIR1/9	Genetic diversity in India and South Korea	[46,47,48]
Synthetic peptides	PvLP1	IgG and IgM, IgG subclass, PBMC proliferation, levels of cytokine and growth factor	[19,32]
	PvLP2	IgG and IgM, IgG subclass, PBMC proliferation, levels of cytokine and growth factor	[19,32]

* Subfamily classification by Lopez and colleagues [35].

## 4. The Role of VIR Proteins in the Spleen 

The spleen is a small organ playing multiple important roles to filter the blood and help defend the body against pathogens. On the other hand, a previous report showed that the spleen may be considered as an accumulation site of *P. vivax*-infected red blood cells resulting chronic infection [55]. Moreover, expression of *vir* genes in the spleen has been detected in monkeys following splenectomy, and the VIR14 protein was found to cytoadhere to cells expressing intercellular adhesion molecule 1 (ICAM-1), a well-known *P. falciparum* receptor [56]. Therefore, it is tempting to speculate that VIR proteins in the spleen play a role in antigenic variation and cytoadherence. However, *P. vivax* VIR proteins have previously been observed in different subcellular localizations, suggesting that they may play a variety of functions; while VIR14-mediated cytoadherence was found to be a function of subfamily C, subfamilies A and D were speculated to play other roles [57]. Therefore, the functions of non-spleen VIR proteins remain unclear.

## 5. The Role of VIR Proteins in Other Organs

Recent ex vivo experiments have shown that *P. vivax* merozoites have a strong preference for invading the immature CD71^+^ subpopulation of reticulocytes, which are typically located in the bone marrow, thus raising the possibility that the bone marrow may house a subpopulation of *P. vivax*-infected RBCs [58]. Moreover, a systematic examination of tissue sequestration during *P. vivax* infection revealed the enrichment of developing sexual stages (gametocytes) and mature replicative stages (schizonts) in the bone marrow and liver relative to the peripheral blood [59]. However, molecules participating in host-parasite interactions during *P. vivax* infection in the bone marrow have not been extensively studied due to difficulties in tissue accessibility. Using ex vivo studies or animal models, such as monkeys, will help to overcome this problem. 

## 6. Conclusions

The subtelomeric *vir* multigene superfamily in *P. vivax* comprises 10–20% of the *P. vivax* haploid genome and is thus likely to play a key role in the pathophysiology of *P. vivax* infection. However, the functions of VIR proteins remain to be fully elucidated. The improved computational analysis offered by the completely sequenced genome of *P. vivax* has helped facilitate molecular studies addressing this essential aspect of *P. vivax* biology. Thus far, several studies have demonstrated the capacity of VIR proteins for mediating cytoadherence and immune response activation. Ultimately, determining the extent to which VIR proteins facilitate prolonged *P. vivax* parasitism, and whether they contribute to severe vivax malaria despite low parasitemia, will guide the development of alternative control strategies against this neglected and non-benign human malaria parasite.

## Figures and Tables

**Table 1:** The *Plasmodium* PIR multigene superfamily.

Species	Family	Total Genes	Location	References
*P. vivax*	*vir*	600–1000	Subtelomeric	[34]
*P. vivax*	*vir*	346	Subtelomeric	[35]
*P. vivax*	*vir*	1220	Subtelomeric	[36]
*P. falciparum*	*var*	60	Subtelomeric and internal	[37]
*P. knowlesi*	*kir*	68	Subtelomeric and internal	[38]
*P. yoelii*	*yir*	795	Subtelomeric	[39]
*P. berghei*	*bir*	200	Subtelomeric	[39]
*P. chabaudi*	*cir*	201	Subtelomeric	[39]

## Data Availability

Not applicable.
